# Mr. Clement, on Vaccine Inoculation

**Published:** 1801-07

**Authors:** W. Clement

**Affiliations:** Surgeon and Apothecary. Shrewsbury


### *To the Editors of the Medical and Phyſical Journal.*

GENTLEMEN,

THE volume of evidence already in favour of the Vaccine Inoculation, may perhaps render it unneceſſary to bring forward any additional proofs of its benign influence.

At

At the period of its introduction into practice, I confess that I was then much prejudiced against it; but to condem a discovery (which has since proved an estimable one) from prejudice, without proof, and without experience, would exhibit a narrowness of mind inconsistent with philosophical enquiry. In the Autumn of 1799, I inoculated three children with Vaccine matter, procured from Dr. Pearson, all of whom had the disease in the usual way. Early the spring following, the Small-pox made its appearance in the neighbourhood; a fair opportunity then occurred to attempt to influence those children, both by inoculation and exposure; which I did, without the least effect, excepting local inflammation. I was likewise, at this time, pretty extensively engaged in inoculating with variolous matter. The comparative mildness between the inoculated Small and Cow-pox, must be acknowledged by all who have practised them, to be much in favour of the latter. In the inoculation of Small-pox many untoward symptoms often arise, both during the period of inoculation and after. Convulsions in the eruptive fever, which, though seldom dangerous, are always alarming, and have in some instances proved fatal. Scrophula is likewise often excited, ophthalmias, and troublesome eruptions of the skin; but what is more generally allowed as an insurmountable objection, is the pressing inducements that frequently occur for inoculating infants before the time of dentition is over; a practice invariably hazardous. Will any professional man then say, that any of those objections, singly or combined, can be offered against the Cow-pox? In the last, and commencement of this year, I have inoculated near *two hundred*, from the age of three weeks and upwards, and not one unpleasant symptom whatever has occurred. During the excessive heat of last summer, the natural Small-pox was very rife, and proved extremely fatal. When but one of a family was attacked with the disease, I frequently got beforehand with it, by immediately inoculating the rest with the Cow-pox; which went through its usual mild course *without eruptions*. In several instances parents had children covered with Small-pox, under the same roof, and often in the same bed, while others were under the influence of the Cow-pox, which proved their perfect security. I hope that it is not necessary to particularize cases, they are so abundant; and so many facts have constantly been adduced, through the medium of your valuable Publication. I have the pleasure to add, that the *Jennerian Inoculation* is universally adopted by the medical gentlemen of this town and neighbourhood. I am, &c.

*Shrewsbury,*

*June 16, 1801*

W. CLEMENT,
Surgeon and Apothecary.

